# Current Trends about Inner Limiting Membrane Peeling in Surgery for Epiretinal Membranes

**DOI:** 10.1155/2015/671905

**Published:** 2015-09-03

**Authors:** Francesco Semeraro, Francesco Morescalchi, Sarah Duse, Elena Gambicorti, Andrea Russo, Ciro Costagliola

**Affiliations:** ^1^Department of Medical and Surgical Specialties, Radiological Specialties and Public Health, Ophthalmology Clinic, University of Brescia, 25123 Brescia, Italy; ^2^Department of Medicine and Health Sciences, University of Molise, 86100 Campobasso, Italy; ^3^I.R.C.C.S. Neuromed, Località Camerelle, Pozzilli, 86077 Isernia, Italy

## Abstract

The inner limiting membrane (ILM) is the basement membrane of the Müller cells and can act as a scaffold for cellular proliferation in the pathophysiology of disorders affecting the vitreomacular interface. The atraumatic removal of the macular ILM has been proposed for treating various forms of tractional maculopathy in particular for macular pucker. In the last decade, the removal of ILM has become a routine practice in the surgery of the epiretinal membranes (ERMs), with good anatomical results. However many recent studies showed that ILM peeling is a procedure that can cause immediate traumatic effects and progressive modification on the underlying inner retinal layers. Moreover, it is unclear whether ILM peeling is helpful to improve vision after surgery for ERM. In this review, we describe the current understanding about ILM peeling and highlight the beneficial and adverse effects associated with this surgical procedure.

## 1. Introduction

Macular distortion and macular edema with the resultant macular dysfunction are the sequelae of epimacular proliferation.

Such proliferation of surface cells is associated with the distortion of both the inner limiting membrane (ILM) and sometimes the outer retinal layers.

The ILM is the basement membrane of Müller cells and is stiffer than the underlying neuroretina that is easily bent or changed in shape.

The ILM provides a support surface to contractile cells acting as a rigid scaffold that transmits the distortion on the underlying retina. Thus, the ILM is closely involved in the pathophysiology of disorders affecting the vitreomacular interface.

The analysis of specimens of ERM after vitrectomy often contains ILM fragments that have been unintentionally removed to treat traction maculopathy [1].

The ILM is the basement membrane between the neuroretina and the vitreous and can act as a scaffold for cellular proliferation in the pathophysiology of disorders affecting the vitreomacular interface.

When ILM is spontaneously separated from the retina in Terson's syndrome, the macula displays no significant reparative fibrosis and maintains excellent visual function during long-term follow-up [2, 3]. These observations showed that removing the ILM is compatible with good visual function, and many surgeons have speculated that the removal of the ILM, which increases the elasticity of the denuded macula, could be exploited in the treatment of diseases that distort the posterior pole [4].

The intentional removal of the macular ILM has indeed greatly improved the anatomical success rate of the surgical treatment of macular hole, and it is a cost-effective option for the treatment of this disease [5, 6]. Therefore, atraumatic ILM peeling has been proposed in the treatment of all forms of traction maculopathy such as ERM, macular hole, vitreomacular traction, myopic foveoschisis, and some forms of chronic diabetic macular edema [7]. However, although the anatomical outcomes are better after ILM peeling, this procedure may potentially cause adverse effects that could affect functional recovery in the medium or long term after surgery.

The introduction of modern Optical Coherence Tomography (OCT) instruments has allowed the identification of anatomical changes that occur months after macular ILM peeling. The formation of irregularities and indentations on the inner surface of the retina, the thinning of the temporal retina, and the thickening of the nasal retina are often evident on OCT frames several months after ILM peeling.

Other aspects like the inner retinal dimpling, firstly called “dissociated optic nerve fiber layer” (DONFL) appearance, may be visible a few weeks after surgery without the use of any sophisticated tools [8].

Finally, the appearance of a transient reduction of the retinal differential light threshold is more marked in cases of ILM removal than in cases in which the ILM is left in place.

Actually, it is not known whether these morphological and functional changes reflect potentially progressive retinal damage.

For most vitreoretinal surgeons, the surgical procedure for treating ERM is well established; however, whether ILM removal is always safe or if it is better to limit this procedure to selected patients remains controversial.

In this review are analyzed the pathogenesis and the treatment of ERM focusing primarily on positive and negative consequences related to ILM peeling.

## 2. Pathophysiology of Müller Cells and ILM

The ILM is a transparent structure that defines the boundary between the retina and the vitreous body. It is composed of the internal expansions of Müller cells and by a meshwork of collagen fibers, glycosaminoglycans, laminin, and fibronectin called the cuticular layer [9].

The ultrastructural analysis of the human retina shows that the ILM appears as a 10 *μ*m thick, homogeneous, periodic acid-Schiff- (PAS-) positive basement membrane; its vitreal surface is smooth and its retinal surface is markedly irregular. The latter surface is made up of Müller cell footplates. An outer dense fibrillar meshwork, the Müller cells basal membrane, and a loose net of fibrils form the cuticular layer [1, 10, 11].

Proceeding from peripheral to central macular retina, the ILM thickness increases up from 0.4 *μ*m to about 1.4 *μ*m.

The ILM is the site of adhesion between the cortical vitreous gel and the retina and is crucial in the pathogenesis of several eye diseases such as idiopathic macular holes, epiretinal macular membrane, and tractional diabetic macular edema.

In physiological retinal processes, Müller cells are able to modulate the concentration of retinal ions through voltage-gated channels, participate in acid-base balance through bicarbonate ions, limit excitatory signals through specific glutamate receptor by a specific reuptake, and provide an adjuvant for metabolic functions (e.g., glycolysis, glycogen metabolism, and oxidative metabolism) of the inner neurosensory retina [12].

Virtually any damage or stimulation on Müller cells can alter retinal function. Every disease of the retina is associated with reactive Müller cell gliosis. Müller cell gliosis is a generic term that reflects the capacity of Müller cells to increase their volume (i.e., hypertrophy) and to proliferate with the aim of supporting the survival of retinal neurons. This reactivity may have deleterious effects on vision, for example, causing intraretinal fibrosis that modifies neuroretinal connections and photoreceptor metabolism and epiretinal fibrosis because the ILM offers a scaffold that permits the adhesion and subsequent proliferation of glial cells [12–14].

Müller cells are the primary support that confers resistance to mechanical stimulation of the retina. Müller cell extensions that blend between the ILM and the external limiting membrane (ELM) exert a major contribution to the biomechanical strength of the retina [15, 16].

The Müller cell footplates that constitute the outer portion of the ILM in the center of the macula form an inverted cone-shaped zone that forms the base of the foveola [17, 18]. This cone acts as a punch, which is the primary point of adhesion between the ILM and ELM over the external segment of foveal cones; this gives the characteristic navel configuration of the fovea. The Müller cells also maintain the nerve fiber bundles of the inner layer of the retina close to each other.

## 3. Composition of Epiretinal Membranes

Epiretinal membranes (ERMs) growing over the macula can result from several pathogenic mechanisms in response to age-related changes such as synchysis and liquefaction in the vitreous humor. They represent a very frequent ocular disease of the elderly. ERMs develop at the vitreomacular interface and are determined by the proliferation of a different type of cells that produce collagen and migrate onto the ILM. These cells gradually form a transparent hypocellular avascular layer and, like all scar tissue, tighten to create tension on the retina, which may bulge and pucker or even cause swelling or macular edema.

The most common form of ERM is idiopathic, which forms in elderly healthy eyes without any other apparent diseases. However, retinal breaks, retinopexy, photocoagulation, inflammation, retinal detachment, and vascular disease (e.g., longstanding central vein occlusion) can also lead to secondary ERM formation.

ERMs are composed of an extracellular matrix (consisting of collagen, laminin, vitronectin, tenascin, thrombospondin, and fibronectin) and of cells. A polymorphous cell population has been found in the membranes: glial cells (e.g., Müller cells, microglia, and fibrous astrocytes); epithelial cells from the retinal pigment epithelium and ciliary body; blood-borne immune cells (e.g., lymphocytes, macrophages, and neutrophils); cells from vitreous fibrocytes (i.e., hyalocytes); and myofibrocytes. Retinal pigment epithelial (RPE) cells also contribute to the formation of ERM in cases of macular holes, retinal tears, and retinal detachment [19–21].

The origin of the epiretinal cells in idiopathic ERM has recently been the subject of numerous studies. The morphologic and histological criteria that were used in the past to classify the cell population of ERM have recently proven to be inadequate because the cells in the vitreous have the capability to undergo striking morphologic changes [22].

The cell population in idiopathic ERM possesses a great variability of immunocytochemical properties because of transdifferentiation in the vitreous [12]. The cells lose contact inhibition and are modified according to the evolutionary phases of the membrane. They are characterized at first to some markers during proliferation and then exposed to other markers during maturation and contraction of the membrane.

Recent studies using proteomic techniques and immunocytochemistry suggest that a large proportion of cells that make up idiopathic ERM is constituted by Müller cells and hyalocytes that undergo transdifferentiation into cells with different characteristics [23–25].

In idiopathic ERM, a large percentage of cells are positive in immunomarkers for Müller cells such as glial fibrillary acid protein (GFAP), cellular retinaldehyde binding protein, vimentin, and Kir4.1; by contrast, immunostaining for pan-cytokeratin is often negative, which predicts little, if any, role of RPE cells in idiopathic ERM [23, 26–28]. Secondary ERM may be formed by RPE cells, fibroblasts, fibrous astrocytes, and cells of blood origin [29].

Müller cells transdifferentiation is characterized by a reduction in cell-specific proteins such as GFAPs and cytokeratins, whereas proteins (e.g., *α*-smooth muscle actin (which is normally not expressed by the cells)) that are involved in motility and proliferation are upregulated. The GFAP content in epiretinal tissues is inversely correlated with clinical contractility [30], which suggests that the transdifferentiation to myofibroblasts increases the capacity of glial cells to generate tractional forces. In addition idiopathic ERMs are positive for immunomarkers typical of Müller cells and hyalocytes.

In addition, the immunomarkers for hyalocytes are positive in ERM. Hyalocytes are of macrophage lineage and have phagocytosis activity and may collaborate with Müller cells as scavengers of debris and apoptotic cells in the pathogenesis of ERM [23, 31]. What activates these cells in idiopathic ERM is not yet known. An important role is probably provided by the mechanical stimulation of the movement of the liquefied vitreous on the retina that results in an immune system response to protect the retina.

The histopathological analysis of surgically removed ERM generally shows two primary types of epimacular proliferation. In type 1, the ERM is in direct contact with the ILM; in type 2, the ERM is laid on a layer of collagen fibers of vitreal origin [23, 32, 33].

This finding underlines two possible theories about ERM pathogenesis. The first and oldest theory is that ERM develops after a cleft in the ILM is created by dynamic vitreous traction on the focal area of adhesion. Through this, Müller cells or other glial cells grow outward from the retina to the inner retinal surface. Müller cell proliferation is aimed at healing the retina (i.e., to heal the ILM break) and at protecting the neuroretinal layers from mechanical stimuli. These reactions create a “conservative gliosis” [34] to protect photoreceptors from apoptosis induced by traction and to resist from the passive movements of the retina [35–37].

This theory seems to be supported by the fact that a group of ERMs, called type II ERMs, are composed of a layer of cells that proliferate directly over the ILM without the interposition of collagen type II [38]. During the peeling of this type of membrane, it is common to simultaneously remove the ILM or its fragments that remain tenaciously adherent to the ERM. However, the presence of an ILM break was never directly demonstrated, even if it could have healed, and this makes it difficult to find a break later in the specimens [39].

The second pattern of ERM, termed type I ERM, is characterized by a layer of collagen between the ILM and the proliferating cells. This pattern seems to underlie a second possibility for ERM formation: a subtle layer of vitreous remains attached to the retina after PVD and this remnant provides a medium for the proliferation of glial cells and hyalocytes [40].

In favor of this hypothesis is the finding of the presence of a premacular oval defect in the detached hyaloid of many patients affected by ERM, which confirms that the rear part of the hyaloid can tear while remaining adherent to the macula [41, 42].

## 4. Etiology and Pathogenesis of Epiretinal Membranes

Posterior vitreous detachment (PVD) is associated with 75–93% of ERMs; it is widely accepted that an anomaly in this process is the primary cause of ERM formation [43, 44]. The modification of the vitreoretinal relationships in aging individuals appears somewhat correlated with a disturbance in collagen metabolism. Posterior vitreous detachment occurs in the same age group that is affected by ERM, and it may precede the onset of ERM symptoms by months or years.

Posterior vitreous detachment commonly occurs bilaterally; the same occurs with ERM, which occurs bilaterally in 20–31% of patients. The unaffected eye has a 2.5 times higher possibility of developing an ERM. This suggests a systemic predisposition to develop an ERM that is probably related to a disturbance of collagen metabolism that is accentuated by age, myopia, and diabetes [45].

Vitreous liquefaction occurs progressively in all eyes with age, and it occurs earlier in myopic eyes than in normal eyes. It appears to be accompanied by a reduced concentration of collagen type IX, which causes the collapse of collagen type II that constitutes the ordinate scaffold of the normal vitreous gel [46, 47]. This significantly reduces the gel volume and increases the liquid volume that creates PVD or posterior vitreoschisis [48]. During eye movement, the shear retinal stress exerted by the vitreous movement on the posterior pole may cause a proliferative cellular reaction and lead to the formation of epiretinal membranes [49–51].

The posterior hyaloid normally adheres to the major superficial retinal vessels, the optic disc, and the macula [52].

At the sites of vitreoretinal attachment, the ILM can become thin and vitreous fibers can adhere directly to Müller cells that support the underlying macular structure and confer the normal shape of the fovea.

Under normal conditions, vitreous fibers exert traction evenly to numerous Müller cells. However, in cases of incomplete PVDs or vitreous shrinkage, a limited area of few Müller cells must support most of the vitreous traction. This may result in chronic mechanical stimulation of the Müller cells and in the local release of inflammatory factors that induce Müller cell gliosis and the breakdown of the blood-retinal barrier [52].

A breakdown of blood-ocular barriers occurs also in cases of ocular inflammation, ischemia, and trauma, all situations associated with ERM formation [52–54]. Vitreous hemorrhage, which sometimes happens in PVD, can be another causative factor that results in the activation of glial cells. In the human vitreous, the presence of biologically active quantities of serum-derived and blood cell-derived cytokines and growth factors, derived by inflammatory blood-borne cells or cell debris, is probably the primary stimulus that triggers and regulates Müller cell process extension and proliferation [55, 56].

The ILM is a reservoir of cytokines. Growth factors regulate the growth and contraction of the ERM. The analysis of human specimens of the ILM and the vitreous associated with a macular hole and the ERM shows several proteins: some, like cytokines and growth factors, are expressed in low abundance; others, such as the heavy and light chains of immunoglobulin G, serum albumin, transferrin, antithrombin III, *α*_1_-antichymotrypsin, hemopexin, *α*_1_-antitrypsin, *α*_2_-HS-glycoprotein, apolipoprotein A-1, transthyretin, apolipoprotein J, fibrinogen *γ* chain, and haptoglobin-1, are expressed in high abundance [57, 58]. Possible mediators for ERM proliferation are basic fibroblast growth factor, nerve growth factor, and glial cell line-derived neurotrophic factor, fibrinogen A, platelet derived growth factors, transforming growth factor *β*1, VEGF (although there are no blood vessels in an ERM), and tumor necrosis factor [59–65].

The contraction of the ERM generates a mechanical stimulus over the ILM that induces further hypertrophy of Müller cells within the retina, thereby causing edema and creating a progressive partially irreversible retinal thickening and photoreceptor disruption. The percentage of loosened photoreceptors can be estimated by the evaluation of the reflectivity of the ELM, the ellipsoid and cone interdigitation zones by OCT. It is a predictive factor for visual acuity recovery after ERM surgery [66, 67].

Even after ERM removal, the total reduction in retinal thickening is not completely possible in longstanding cases of glial scar. Long-term vitreoretinal traction, especially if it disrupts the blood-brain barrier and causes macular edema, is more likely to create significant intraretinal Müller cell proliferation and irreversible functional and structural disruption of the neural retina that does not permit good visual recovery after surgery.

A suggestive theory to clarify the pathogenesis of tractional macular diseases has been proposed by Sebag [68]. He suggested that the phenomenon of vitreoschisis is the basis of most tractional diseases of the macula. According to this theory, PVD is a physiological phenomenon of aging caused by two main changes in the vitreous humor: the liquefaction of the central part of the vitreous and the weakening of adhesions between the retina and the posterior hyaloid. In most patients, PVD occurs in a physiological manner with complete separation of the posterior hyaloid from the retina without collagen remnants on the ILM surface. In pathological PVD, the weakening of the adhesion between the posterior hyaloid and the retina does not occur. The liquefaction of the central vitreous creates a dynamic traction on the fibers of the cortical vitreous adherent to the posterior hyaloid that are arranged in layers like onion bracts. This causes a cleft in the thickness of the cortical vitreous and creates vitreoschisis, in which the most peripheral layer of the posterior hyaloid remains attached to the retina and separates from the other layers [69].

Hyalocytes within the vitreous cortex remnants remain on the inner retinal surface after PVD. In this situation, an unknown stimulus induces the hyalocytes to stimulate the intraretinal Müller cells to proliferate on a layer of vitreal collagen fibers. Epiretinal membranes, once formed, tend to progress even when the original inciting stimuli are decreased or eliminated, because the cells within the membranes can produce growth factors and cytokines that recruit other cells and stimulate their proliferation.

It has been suggested that a primary mechanism that leads to ERM enlargement is phagocytosis of blood-borne substances and cell debris, which adhere to the vitreal surface of the retina by Müller cell processes that extend through holes in the basement membrane of the ILM [19]. The proliferation of these cells may paste the ILM to the vitreous and to the ERM. In this situation, ERM removal cannot be performed without removing the ILM at the same time.

Müller cells and hyalocytes can proliferate on the layer of vitreous fibers that remain on the retina and form the ERM; they also can colonize all areas where there is a vitreous-liquid interface [70].

When the vitreomacular traction is stronger than the mechanical resistance of the center of the foveola, foveal integrity may be damaged by vitreous movement or by surgery. This harmful event can occur during surgery, vitreofoveal traction, or myopic traction maculopathy and lead to the formation of a macular hole [71].

The thickness and the size of the area of adhesion between the retina and the hyaloid explain why different tractional macular pathologies exist. If the area of vitreomacular traction is focal and anteroposterior, it may result in vitreofoveal or vitreomacular traction syndrome; if the area of traction is focal and centered on the edge of the foveola, it may form a macular hole; and if the area of traction is wide and spreads across the macula, it may form a macular pucker.

In high myopia, PVD is often complicated by large sheets of residual cortical vitreous that remain attached over the inner surface of the retina and may subsequently contract, thereby giving rise to tractional vitreoretinal diseases. If the thickness of the vitreous that remains adherent to the retina is remarkable, this may result in myopic vitreous macular traction syndrome, which is also called “myopic foveoschisis.”

Finally retinal breaks, retinopexy, photocoagulation, inflammation, retinal detachment, vascular disease, and, more rarely, retinitis pigmentosa (over the peripapillary retina), hemorrhagic glaucoma, Terson's syndrome, Eales disease, and Coats disease may cause secondary ERM [53, 72–76].

## 5. Epidemiology and Treatment of Epiretinal Membranes

ERMs are also called “cellophane maculopathy,” “macular puckers,” “surface-wrinkling retinopathy,” “epiretinal gliosis,” and “premacular fibrosis.” The prevalence of idiopathic ERM depends primarily on the age of patients: they may occur in 2% of people under 60 years of age, but the prevalence increases to 12–20% after the age of 70 years [45] and is bilateral in 10–20% of patients [77].

Approximately 30 years ago, Machemer introduced vitrectomy for the treatment of macular pucker. Since it was first proposed, the technique of removing the ERM as a single piece has not changed significantly, and the removal of the pucker is always performed without the ILM peeling with substantially favorable results [78–80].

Surgery is recommended if the blurred vision or the distortions are severe enough to interfere with binocular vision or daily living. Many case reports achieved good results simply by removing the ERM and reduced metamorphopsia and improved visual acuity in 70–90% of patients with a mean improvement in vision by 2 or more Snellen lines [38, 81–84]. The visual acuity improvement continued for the next 6–8 months and the best final visual acuity may be obtained after 1 year [80].

Surgery for macular pucker allows the recovery of approximately one-half of the visual acuity that had been lost, and visual recovery is greater if the preoperative visual acuity is lower. However, the probability to regain vision after surgery is increased in patients with a preoperative visual acuity of 0.25 or better; patients with better baseline visual acuity can get a full visual recovery [85].

Complete recovery of vision is rare in patients with longstanding ERMs, and retinal thickness and the macular profile rarely return to normal. Thus, early surgery is likely to decrease the risk of developing irreversible macular damage [86].

Despite seemingly adequate and complete removal of the ERM, some patients continue afterwards to complain of blurred vision, slight metamorphopsia, or distortion [87]. Furthermore, ERMs may form again months after apparently successful epimacular proliferation removal; it is estimated that up to 16.5% of patients may have ERM recurrence after surgery. This phenomenon requires a repetition of the surgery for the pucker in 3–6% of patients [79, 80, 88]. Patients affected by secondary ERM and young patients have more recurrences and, in this category of patients, final visual outcome is usually less satisfactory [89].

## 6. ILM Peeling in Surgery of Epiretinal Membranes

Until 1990, the ILM was considered an integral part of the retina, and vitreoretinal surgeons did not think that it could be removed without causing damage to vision.

The reports of cases of spontaneous separation of the ILM in Terson's syndrome, which resulted in no significant reparative fibrosis and good visual prognosis after surgery, have attracted the attention of vitreoretinal surgeons; this phenomenon showed the possibility of removing the ILM to release vitreoretinal tractions [2, 3]. Furthermore, the histological examination of the removed ERM shows that, in 40–60% of patients, the ILM and ERM are so adherent they are often removed together at the same time, thereby confirming the hypothesis that these 2 membranes are strictly linked in causing epiretinal puckering [90, 91].

On the other hand, the ultrastructural examination of ILM specimens, which were removed after ERM peeling, demonstrates the presence of microscopic ERM remnants that persist over the ILM in almost one-half of the patients [92–94]. These observations highlight that the conventional way of peeling the ERM leaves fragments of cells behind on the ILM and that these residual tissues could form the islands of reproliferation [91].

ILM removal provides the certainty of having removed all cells that produce collagen above the retina, thereby eliminating the scaffold for proliferative cells such as transdifferentiated Müller cells and myofibroblasts, which are the prevailing type of cells in recurrent ERM [95]. In addition, this procedure ensures that all adhesions that corrugate the inner retina have been released, because the ILM can stiffen and thicken in the process of ERM formation. Thus, in the early 90s the first studies appeared in which the ILM was removed during ERM surgery [96].

The simultaneous separation of ERM and ILM apparently does not cause adverse effects on vision. In some studies, the visual acuity indeed appeared better in patients in whom large portions of the ILM were removed with the ERM [97]. For example, Bovey et al., in their prospective case-control trial, reported a final visual gain, at 21 months of follow-up, of 3.1 lines when ILM peeling is performed and 0.9 lines when ILM is not removed. However, in one retrospective study conducted on 41 patients, the ILM removal was reportedly correlated with worse visual functionality [98].

In 2000, the introduction of vital dyes in vitreoretinal surgery revealed other findings that previously had not been noticed. The simple removal of the ERM may also partially separate the ILM from the retina that after several months may contract again causing residual traction and retinal striae. By performing ILM peeling, the retinal striae are more likely to disappear or flatten [96].

In 2003, a randomized pilot study showed that peeling of the ILM during ERM surgery may not have a deleterious effect [99]. ILM peeling was also found superior in resolving cystoid macular edema due to epiretinal traction, which disappeared in 90% of patients, compared to 44% of patients who had undergone removal of the ERM only [86].

Thus, the simultaneous removal of ERM, followed by ILM peeling, has become a widely approved procedure in vitreoretinal surgery. In a few years, the number of surgeons routinely performing this procedure has widely risen.

Numerous studies confirmed that ILM removal during surgery for ERM is associated with better anatomical improvement, better final vision, and a lower risk of recurrent epimacular membranes [86, 94, 97, 99–104].

From a surgical point of view, the peeling of this ERM type I is easier because a cleavage plane exists that is a collagenous layer interspersed between the ILM and the cells. However, by removing only the first membrane, a certain amount of collagen and cells remains over the ILM. A histological study has verified that the removal of only the ERM can leave on the ILM surface up to 20% of the cells that compose epimacular proliferation in two-thirds of patients [33].

Thus, a second membrane, made by the ILM with residues of collagen, should be removed to ensure eliminating all tangential traction over the retina and to avoid ERM recurrence [91].

In type II ERM, ILM and ERM are so adherent that they often are separated at the same time during surgery [88, 90, 91]. The release of the epiretinal traction inhibits the stimulus for hypertrophy of Müller cells within the retina but appears to not completely inhibit the growth of these cells onto the surface of the ILM where they can reform a new glial scar [36].

The omission of the removal of the ILM could not inhibit the growth of glial cells above and below the ILM where they can reform a new macular pucker. Thus, ILM and ERM are considered of the same pathology and should be removed together [105, 106].

## 7. Vital Dyes to Highlight the ILM

The difficulty of distinguishing the ILM from underlying structures makes ILM peeling a challenging maneuver. Failing to distinguish details of ILM can lead the surgeon to cause damage to the nerve fibers, extend the time of surgery, and lead to increased inflammation with subsequent responsive macular edema. To facilitate ILM clear identification the use of vital dyes has been introduced since 2000 and is currently used by the vast majority of vitreoretinal surgeons.

The first among these dyes was indocyanine green (ICG) that at the concentration of 5 mg/mL (0.5%) provides a stark contrast between the stained and the unstained ILM [107–109].

Early clinical studies with the use of ICG have reported good anatomical and functional results [110–113].

However, subsequent studies have found that intraocular ICG can cause toxic effects to both the neuroretina and pigmented epithelium [114, 115] and this could compromise the functional success of the surgery [116].

A direct toxicity on retinal glial, EPR, and ganglion cells has been highlighted in in vivo and in vitro studies [114, 115, 117, 118].

The morphological examination of samples of ILM after the use of ICG demonstrated that the cleavage plane of the membrane is deepened and its removal also removes layers of Müller cells [119]. Far more cellular debris on the retinal side of the ILM were seen in the ICG-stained in comparison with the unstained specimens. ICG seems to be toxic also for the hypoosmolarity of the injected solution and for a phototoxic effect triggered by natural light or by the endoilluminator [117, 118].

It was also discovered that ICG could persist on the inner retina for many months after surgery so the phototoxic effect could last a long time [120].

Other vital dyes were later introduced to replace the ICG: Trypan Blue 0.15%, Brilliant Blue, triamcinolone acetonide, and very recently Acid Violet 17.

Trypan Blue is not specific for the ILM but stains sufficiently the inner retinal surface and allows a useful contrast between the colored surface and the underlying unstained layers.

It appears to be less toxic than ICG, as shown by studies that highlight the best functional results and the lower incidence of central scotoma in groups of patients that were treated by vitrectomy with ILM peeling and stained with Trypan Blue versus ICG [121].

Brilliant Blue G is another vital dye that has been introduced after the Trypan Blue. It has a good safety profile, provides significant anatomical and functional postoperative results [122], and has the peculiar characteristic of staining specifically the ILM and not the rest of the retina as well as ICG.

Triamcinolone acetonide (TA) is a synthetic glucocorticoid that can be formulated to intraocular use. It has the consistency of a whitish powder that forms a deposit on the retinal surface. It can be used to distinguish the epiretinal membranes and the posterior hyaloid from the inner retina and the ILM from the underlying retinal layers. It has the major drawback of dirtying the tip of the instruments and being absolutely a nonselective dye.

TA is considered safe [123]; however, studies exist that highlight long-term toxicity when it is used in high concentrations like transient but consistent intraocular pressure elevation and in very few cases acute endophthalmitis [124]. In animal species, some toxic effects have been shown on RPE cells, retinal Müller glial cells, and retinal neurosensory cells [125].

Finally, very recently the use of another vital dye has been introduced: the Acid Violet 17 that is specific to the ILM and allows its clear intraoperative visualization. Acid Violet 17 was safe for the retinal tissue at concentrations of 0.25 and 0.50 g/L after intravitreous injection; however further studies are required to investigate its long-term safety [126].

## 8. Concerns about ILM Peeling in Epiretinal Membrane Surgery

After ERM surgery retinal thickness as well as the macular profile rarely returns to normal.

The partial recovery of macular morphology is due to the chronic deformation exerted by the ERM that caused hypertrophy of Müller cells whose ramifications tend to fill all the empty spaces previously occupied by other degenerated neurons. Also the intraretinal edema creates an irreversible alteration of the retinal structure and probably of the retinal function [79, 80, 88].

Similarly, complete recovery of vision is rare and mostly dependent on visual acuity before surgery. ERM surgery allows recovery of approximately one-half of the visual acuity that has been lost [96].

Mechanical injury to the neurosensory retina during ERM and ILM peeling could have a role in partial postsurgical recovery of vision.

Cystoid macular edema is a disappointing and relatively common complication. Surgical traction on Müller cells may induce damage to their function and gliosis of the ELM with subsequent accumulation of proteins and material over its inner side, thereby causing the cystoid macular edema [98, 127].

A recent study showed that the glial proliferation involves also the retina under the ILM [128]. The authors observed that ILM removal is more difficult during ERM surgery than in macular hole. They detected glial and/or neuronal cells on the retinal surface of the ILM in 32% of the macular hole-ILM specimens and in 65% of the ILMs peeled after ERM removal; this difference was significant. These findings suggest that ERM may be associated with sub-ILM fibrosis that alters the plane of separation during ILM peeling and that a possible loss of superficial nerve fibers is to be expected after ILM peeling in some patients. In fact, OCT examination shows a thinning of the retinal nerve fiber layer (RNFL) after surgery.

The ERM may have a significant intraretinal component under the ILM. This indicates that this disease may affect the entire thickness of the retina, not just the inner layer. The proliferation of these cells may paste the inner retinal layers to the ERM; in that case, ERM removal cannot be performed without ILM removal at the same time.

When this adhesion is particularly strong, the center of the foveola may be damaged by surgery. This event may be harmful in case of small but tenacious adherence of the ERM to the center of the macula. If the macula is thickened, that is, in case of vitreofoveal tractions or in myopic traction maculopathies, the surgical traction may cause the formation of a macular hole [71].

ILM removal may also result in glial apoptosis due to removal of Müller cell plates and may be responsible of weakening of the retina, thereby leading to eccentric retinal hole development. The etiology of these holes may be due by contracture of the remaining epiretinal proliferation, thereby causing expansion of a previously undetectable iatrogenic defect [129]. After ERM combined with ILM peeling, the foveal depression rarely forms again.

Thickening of macula without foveal depression has been found in 84.2% of patients of ILM-peeled eyes, compared to 42.9% of patients with unpeeled eyes; a normal foveal contour with a foveal depression has been found in only 15.8% of ILM-peeled eyes, compared to 57.1% of unpeeled eyes [105]. ILM peeling could damage the Müller cell footplates that form the inverted cone scaffold that gives the navel shape to the fovea. The fovea remains virtually without any lateral structural support. Thus, some authors recommend leaving the ILM just above the fovea [18].

Visual recovery after surgery ERM can also be achieved without combining ILM peeling. Many works of comparison found no functional difference between the groups in combination and without with ILM peeling [88, 130].

Tadayoni et al. first reported anatomical damage after ILM peeling and first described a peculiar macular appearance, called “dissociated optic nerve fiber layer” (DONFL), which appeared 1–3 months after ERM surgery [8].

Blue light autofluorescence and infrared reflectance imaging may also provide evidence for arcuate striae formed by nerve fibers that radiated from the macula to the papilla in an arcuate fashion. Their appearance reflects a swelling of nerve fiber bundles and is visible as early as 1 week until 1 month postoperatively and disappears after 2 months [131]. In fact, the retinal nerve fiber layer (RNFL) appearance with the OCT is thickened in the first month after surgery [132]. After a short period, the RNFL displays a tendency to decrease from the third month and become progressively more apparent many months postoperatively. The RNFL decreases especially in the temporal quadrant and becomes thinner than before surgery [132–134]. At last, the appearance of the macula after 3–6 months has often nicks and dimples in the inner surface [132, 135].

At first, the use of ICG was held responsible for toxic and mechanical damage to the inner retina leading to the thinning of the RNFL [114, 115]. However, even with the use of other vital dyes such as Trypan Blue, the reduction of RNFL thickness as well as the phenomenon of DONFL and arcuate swelling of the nerve fiber layer occurs [136].

Finally, a possible side effect that may be related to ILM peeling surgical procedure is an ipo/atrophic modification in macular region. Baba et al. reported a partial macular hypotrophy with a reduction in the thicknesses of the inner retina and ganglion cell complex [135].

In most cases, however, with the tools available today there is not obvious demonstrable functional damage caused by these anatomical macular modifications.

Most patients do not show any symptomatic visual field defect, and most patients after ILM peeling have an improvement in their vision and reading speed [137, 138].

## 9. Discussion and Conclusions

The ILM is the boundary that establishes the contact and communication point of two compartments: the retina and vitreous. The inner part of ILM has a living boundary formed by the footplate processes of Müller cells. These glial cells regulate retinal homeostasis and functionality; however, they also constitute a scaffold for the correct positioning of all neural cells with a particular importance in maintaining the shape of the fovea.

Thus, the ILM is the pivot on which vitreal tractions spread throughout the retina. The tractions from the inner surface of the retina are transmitted to the whole retina through the Müller cell network and through the ELM, which then stretches the photoreceptors. The shear stress generated by the movement of the liquefied vitreous on abnormal vitreomacular adherence triggers specific natural inflammatory reactions. The expression of contractile proteins by these cells transforms Müller cells into microfibroblasts.

Müller cells react to mechanical and hypoxic stimuli by hypertrophy to resist and protect the neuroretinal layers from traction (i.e., passive movements induced by traction) and to protect photoreceptors from apoptosis. The Müller cells reaction however is self-maintained by a vicious circle in which hypertrophy is followed by transdifferentiation, proliferation, and contraction. The vitreous initiates the pathology that the retinal cells worsen. Epiretinal membranes, once formed, tend to progress, even when the original inciting stimuli are decreased or eliminated, because the cells within the membranes can produce growth factors and cytokines that recruit other cells and stimulate their proliferation.

Pathological vitreoretinal adhesion on the ILM offers different surfaces on which transdifferentiated glial cells migrate and thereby configures different aspects of traction maculopathies.

Vitrectomy is performed to release the pathological influence of the vitreous on the retina and is useful in restoring the normal anatomical shape of the macula and improving visual acuity. The removal of the ILM has been the major advance in vitrectomy in the past 15 years. On the other hand, the surgical technique of ILM peeling may unintentionally injure the underlying retina. It often depends on the degree of adherence of the epiretinal membrane.

Traction during ILM peeling could lead to a retinoschisis or to accidental interruption of the intraretinal neural network or the nerve fiber bundle. In addition to the damage closely associated with the surgical technique, recent findings have revealed adverse effects related only to the peeling of the ILM, including damage to the tropism of the Müller cells, a decrease in foveal retinal sensitivity, and alteration of the b-wave of electroretinograms [139, 140].

In conclusion, it is not possible with the present knowledge to confidently choose whether peeling the ILM in ERM surgery is associated with an improvement in vision.

No sufficiently large randomized clinical trials (RCTs) on this topic are available. Further studies are desirable to increase our knowledge on the physiology of Müller cells, which are closely related to the physiology of the ILM.

Studies are underway on supplying astrocytes as a strategy that will inhibit the exaggerated response of glial cells to mechanical and ischemic stimuli in order to restore the physiological network of capillaries in avascular retina areas. In addition, the delivery of recombinant pigment epithelium-derived factor may allow the recovery of Müller cells and thus creates favorable conditions for the survival of retinal cells in the loss of their homeostasis [141].

Moreover, the mechanism and the severity of the traction on the inner retina can vary from patient to patient, depending on the amount of cells in the ERM (i.e., sparse cellular proliferation or dense cellular proliferation). The latter group (i.e., patients with dense cellular proliferation) may be associated with a higher chance of surgical difficulty during ILM peeling.

Surgeons using vital dyes must assess the presence of cortical vitreous on the ILM after having first removed the ERM. If there is residual vitreous and it is not possible to remove it, peeling of the ILM is indicated. Surgeons must also consider whether the ILM is intact or damaged after ERM removal. If the ILM is not damaged, the surgeon may decide to leave it; if it is damaged or responsible for retinal striae, probably it should be removed [142]. The surgeon must preserve the eye from ILM removal in cases of retinal thinning for circulatory or metabolic disorders and in case of glaucoma. Additional surgical experiences and further functional studies must be conducted to determine if it is safe to leave a portion of the ILM in front of the fovea.
